# Polysubstance Use and Social Sequelae in Women of Reproductive Age with Recent Marijuana Use

**DOI:** 10.3390/pharmacy13040092

**Published:** 2025-07-02

**Authors:** Sean Hyungwoo Kim, Hua Min, Hong Xue, Panagiota Kitsantas

**Affiliations:** 1 Department of Biopharmaceutics and Pharmacogenomics, Bernard J. Dunn School of Pharmacy, Shenandoah University, 1775 N. Sector Court, Winchester, VA 22601, USA; skim3@su.edu; 2 Department of Health Administration and Policy, College of Public Health, George Mason University, 4400 University Drive, Fairfax, VA 22030, USA; hmin3@gmu.edu (H.M.); hxue4@gmu.edu (H.X.)

**Keywords:** women of reproductive age, marijuana, polysubstance use, social sequelae, National Survey on Drug Use and Health

## Abstract

Polysubstance use (PSU) involving marijuana among women of reproductive age (WRA) is linked to psychosocial harm, yet research on the combined effects of marijuana with stimulants, opioids, tobacco, and binge drinking remains limited. The purpose of this study was to assess the prevalence of PSU in WRA with past month MJ use and examine the association between PSU status and social sequelae, including getting in trouble with the law, relationship difficulty with others, and lower achievement in job or educational settings, in this group of women. We used data from the United States 2015–2019 National Survey on Drug Use and Health, which included 11,895 non-institutionalized WRA (18–44 years old) with reported use of MJ in the past month. Descriptive statistics, chi-squared tests, and multivariable logistic regression analyses were conducted to describe the sample and assess associations between PSU and social sequelae. Nearly 4.5% of the women who used MJ in the past month had experienced social sequelae regardless of PSU status. Women who used three or more substances along with MJ had the highest adjusted odds (AOR = 3.18, 95% CI 1.90, 5.31) of social sequelae relative to monosubstance MJ users. Concurrent use of multiple substances significantly increased the likelihood of social sequelae among women MJ users.

## 1. Introduction

Polysubstance use (PSU), which is defined as the ingestion of more than one drug of abuse within a defined period [1], may include a wide spectrum of illicit substances such as marijuana (MJ), psychoactive stimulants (cocaine, crack, methamphetamine), opioids (including heroin), licit substances (e.g., alcohol, tobacco/cigarette), or misuse of prescription opioids or psychoactive stimulants that contain amphetamine-related compounds. *Cannabis sativa* or MJ is the illicit drug under the United States (US) federal law most commonly used during pregnancy with self-reported prevalence of MJ use that ranges from 2 to 5% in most studies [2]. Research findings on PSU among pregnant women show the prevalence of past month MJ use at 3.9% and 7.6% in non-pregnant women of reproductive age (WRA) along with past month use of tobacco (3.2% in pregnant women, 2.3% in non-pregnant women), alcohol binge drinking (four or more drinks per occasion in 30 days, 1.9% in pregnant women, 2.9% in non-pregnant women), and other illicit drugs (2.7% in pregnant, 2.7% in non-pregnant women), including cocaine and heroin [3].

MJ use is associated with PSU or problematic misuse with other substances including alcohol, tobacco, and other illicit drugs including opioids and psychoactive stimulants where overlapping effects are expected [4,5]. There are risks associated with PSU or concurrent use of multiple substances with MJ within a defined period (i.e., past month or past year) including dangerous patterns of substance use (i.e., addiction, overdose), physical harm (i.e., self-harm or suicidal behavior), psychosocial harm (i.e., depressive episodes, social sequelae such as getting in trouble with the law, relationship difficulty with others), and lower educational or occupational achievements [6,7]. In comparison to those who used a single substance (monosubstance users), polysubstance users, or those who consume two or more substances, have increased risk in dangerous patterns of substance use and psychosocial outcomes [6]. Considerable evidence suggests that MJ use is associated with reduced educational attainment (i.e., school dropout), higher chance of welfare dependence, unemployment, criminal behavior, and poor outcomes on a variety of life satisfaction and achievement measures [8,9,10,11].

Although several studies have examined the effects of PSU on psychosocial outcomes within the general population as well as prevalence of PSU in WRA including MJ use, no study has specifically assessed associations between PSU status and psychosocial harm including social sequelae such as (1) getting in trouble with the law; (2) relationship difficulty with family or friends; and (3) lower achievement in occupational or educational setting in WRA who used MJ in the past month. The WRA constitute a potentially vulnerable population, given the risks to both women themselves, the fetus, if pregnant, and infant during breastfeeding [4,12]. In sum, social sequelae attributable to MJ with concurrent use of other substances highlight the need for a study to better understand PSU patterns in this vulnerable population.

The purpose of this study was to (1) assess the prevalence of PSU or concurrent use of psychoactive stimulants (cocaine, crack, methamphetamine, and misuse of prescription stimulants that contain amphetamine-related compounds), opioids (including heroin), tobacco, or alcohol binge drinking in WRA (18–44 years old) who reported past month MJ use and (2) examine the association between PSU status and social sequelae, including getting in trouble with the law, relationship difficulty with others, and lower achievement in job or educational settings in this group of women.

## 2. Materials and Methods

### 2.1. Data and Sample

Data from the National Survey on Drug Use and Health (NSDUH) for the years 2015–2019 were utilized for this study. The NSDUH is a US nationally representative cross-sectional survey annually conducted by the Substance Abuse and Mental Health Services Administration (SAMHSA). Self-reported substance use and social sequelae measures due to MJ were collected in the survey assessing prevalence, frequency of substance use, social sequelae, and other health-related conditions in the US civilian and non-institutionalized populations aged 12 years and older via audio computer-assisted self-interviewing or ACASI reporting [13].

From the 2015–2019 NSDUH dataset, the analytic sample included 11,895 adult WRA from 18 to 44 years old who used MJ in the past month. Exclusion criteria included young adolescents (12–17 years old), females older than 44 years of age and respondents who identified themselves as male. Despite the availability of the most up-to-date NSDUH data, from 2020 to 2023, only data up to 2019 were analyzed due to unavailability of MJ related social sequelae data in the NSDUH after the year 2019.

### 2.2. Measures

#### 2.2.1. Polysubstance Use Status

Polysubstance MJ users (PMU) were identified based on their responses to the following questionnaire on four classes of substances: (1) “Cigarette/tobacco use in the past 30 days?”; (2) “Binge alcohol use in the past 30 days?” Binge alcohol use in the past 30 days were defined as drinking four or more drinks on the same occasion on at least 1 day in the past 30 days; (3) “Opioid use in the past 30 days?”; (4) Concurrent use of psychoactive stimulants with MJ were determined by combining three yes/no questions on prescription stimulant use (“Prescription stimulant use in the past 30 days?”), cocaine including crack use (“Cocaine including crack use in the past 30 days?”), and methamphetamine use (“Methamphetamine use in the past 30 days?”). The rationale for selecting the aforementioned four substances as candidates for PSU or concurrent use with MJ were adopted from the 2015 US CDC study on prevalence and patterns of MJ use among pregnant and nonpregnant WRA [3] and a Swedish study that suggests that the most common illicit substance in combination with MJ included psychoactive stimulants (amphetamines), and heroin [14].

Several measures were created to assess the prevalence of PSU in WRA who used MJ in the past month and examine the association with social sequelae. PSU status was measured based on binary variables (yes/no) for all substances considered in this study. An overall PSU status variable was created with two levels, PMU versus only women who used MJ without any other substances or monosubstance MJ users (MMU). A four-level PSU status variables was created to assess its impact on psychosocial harm: (1) women who engage in MJ use only in the past month without four other substances including tobacco/cigarette, alcohol binge drinking, psychoactive stimulants, and opioids were classified as MMU; (2) women who engage in MJ and one of the four other substances in the past month were classified as ‘Polysubstance Marijuana Users I’ or PMU I; (3) women who engage in MJ and two of the four other substances in the past month were classified as ‘Polysubstance Marijuana Users II’ or PMU II; and (4) women who engage in MJ and three or more other substances in the past month were classified as ‘Polysubstance Marijuana Users III’ or PMU III.

Based on our analyses, there were 2770 women who were classified as MMU if women only used MJ in the past month without using any of the four substances including tobacco, alcohol binge drinking, psychoactive stimulants, and opioids. There were 5129 women who were classified as PMU I if women used MJ with one of the four other substances in the past month. There were 3142 women who were classified as PMU II if women used MJ with two of the four other substances in the past month. Lastly, there were 854 women who were classified as PMU III if women used MJ with three or more substances in the past month.

#### 2.2.2. Social Sequelae of Marijuana Use in Women of Reproductive Age

Women’s social sequelae attributable to MJ use were assessed based on the following three NSDUH yes/no questions: “During the past 12 months, did using marijuana or hashish cause you to have serious problems either at home, work, or school?”, “During the past 12 months, did using marijuana or hashish cause you to do things that repeatedly got you in trouble with the law?”, “During the past 12 months, did you have any problems with family or friends that were probably caused by your use of marijuana or hashish?”. Based on the responses to these three questions, women were classified into two groups, namely ‘yes social sequelae’ group (presence of social sequelae caused by MJ) versus ‘no social sequelae’ group (absence of social sequelae caused by MJ). The final analytic social sequelae variable included 623 women (5.24%) who answered ‘yes’ to any of those three questions. Specifically, 378 women answered ‘yes’ to the “during the past 12 months, did using marijuana or hashish cause you to have serious problems either at home, work, or school?” question. In addition, 125 women answered ‘yes’ to the “during the past 12 months, did using marijuana or hashish cause you to do things that repeatedly got you in trouble with the law?”. Moreover, 340 women answered ‘yes’ to the “during the past 12 months, did you have any problems with family or friends that were probably caused by your use of marijuana or hashish?”. It is important to note that the same women could answer ‘yes’ to two or more social sequelae related questions. Women who answered ‘no’ to all three social sequelae related questions were 11,176 women (93.96%). Lastly, there were 96 women (0.81%) who did not report their social sequelae status (missing data). To current knowledge, this type of classification is unique and has not been used in prior studies using NSDUH datasets.

#### 2.2.3. Sociodemographic Factors

Demographic characteristics included adult WRA (18–25 years old and 26–44 years old), race/ethnicity (White non-Hispanic, Black non-Hispanic, Asian/other non-Hispanic, and Hispanic), education level (no high school education, high school education or GED, some college/college graduate), and employment status (employed full-time, employed part-time, unemployed, and other). Other measures included no health insurance or health insurance gap (yes/no to a question on whether respondents did not have health insurance at some point in the past 12 months).

#### 2.2.4. Self-Reported Health

A past year major depressive episode (MDE) and self-reported overall health were assessed as well. Women’s past year MDE status was measured based on the presence or absence of a past year MDE (binary ‘yes’ or ‘no’ variable). Self-reported overall health status was assessed based on the following question: “How would you say your health, in general, is?” and this variable was categorized as “Excellent/Very Good, Good, and Fair/Poor”.

## 3. Statistical Analysis

Descriptive analyses were conducted to describe the sample characteristics of adult WRA who used MJ in the past month as well as PSU MJ users. The prevalence of PSU status and social sequelae in this population were determined. Bivariate analyses using the chi-squared test were conducted to examine any significant associations between PSU status and social sequelae among women who reported MJ use in the past month. A binary logistic regression model was built to examine the size and direction of the associations between PSU status (MMU as reference group) and social sequelae attributable to MJ use after adjusting for sociodemographic factors and self-reported health status. The data were weighed to account for the complex design of the NSDUH. Stata/SE version 16.1 statistical software was utilized in the analyses (StataCorp, 2019. College Station, TX, USA).

## 4. Results

### 4.1. Sample Characteristics

Table 1 shows sample characteristics in WRA (18–44 years old) with past month use of MJ stratified by PSU status. Nearly 4.45% (95% CI: 3.98, 4.98) of the past month MJ women users experienced social sequelae. There was a notable increasing trend in the prevalence of social sequelae with PSU or concurrent use of other substances with MJ including alcohol binge drinking, opioids, psychoactive stimulants, or tobacco/cigarettes. The prevalence of social sequelae among MMU was 3.81% (95% CI: 3.02, 4.81) which is significantly lower compared to women involved in PSU or concurrent use of MJ with two forms of substance (PMU II) with 4.96% (95% CI: 3.94, 6.18). Lastly, compared to women in the MMU group, women involved in PSU or concurrent use of MJ with three or more forms of substance (PMU III) had the highest prevalence of social sequelae with 10.18% (95% CI: 7.63, 13.45).

The majority WRA were White non-Hispanic (61.66%, 95% CI: 60.14, 63.16), who had attained some college education or were college graduates (67.99%, 95% CI: 66.91, 69.06), did not have any health insurance gap in the past year (88.99%, 95% CI: 88.07, 89.85), and were aged 26–44 years old (58.35%, 95% CI: 57.34, 59.35). Nearly 22.59% (95% CI: 21.63, 23.56) of the WRA with recent MJ use experienced MDE in the past year. Similarly, the prevalence of MDE showed an increasing trend with PSU status; concurrent use of other substances with MJ was associated with increased prevalence of MDE. Women involved in PSU with two forms of substance with MJ (PMU II) had higher prevalence (24.93%, 95% CI: 22.94, 27.03) of MDE while women involved in PSU with three or more forms (PMU III) of substance with MJ had the highest prevalence (29.49%, 95% CI: 25.45, 33.88) of MDE. Having a health insurance gap in the past year, being White non-Hispanic, and in Fair/Poor self-reported overall health were significantly associated with higher prevalence of PSU with MJ.

### 4.2. Social Sequelae in Women of Reproductive Age (18–44 Years Old)

**Figure 1:**
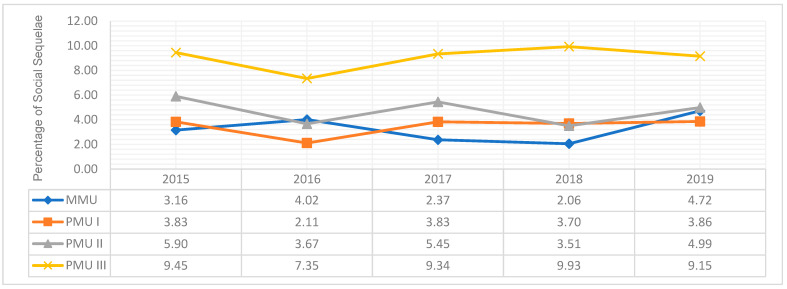
**Social Sequelae in Women of Reproductive Age (18–44 years old) by Polysubstance Use Status From 2015 to 2019. MMU** refers to women involved in monosubstance use of marijuana only in the past month without use of other substances (alcohol binge drinking, opioids, psychoactive stimulants, and tobacco). PMU I refers to women involved in polysubstance use of marijuana in the past month with one other substance (alcohol binge drinking, opioids, psychoactive stimulants, or tobacco). PMU II refers to women involved in polysubstance use of marijuana in the past month with two other substances (alcohol binge drinking, opioids, psychoactive stimulants, or tobacco). PMU III refers to women involved in polysubstance use of marijuana in the past month with three or more other substances (alcohol binge drinking, opioids, psychoactive stimulants, or tobacco).

Figure 1 displays the prevalence of social sequelae by PSU status for five time periods (year), namely 2015, 2016, 2017, 2018, 2019. Overall, women involved in PSU or concurrent use of MJ with three or more substances (PMU III) in the past month had the highest prevalence of social sequelae. Between 2015 and 2019, women in the PMU III group had the highest prevalence of social sequelae that ranged from 7.35% to 9.93%. Notable increases in PSU with concurrent MJ use were observed since 2016 with the exception of MMU which shows an increase after 2018.

### 4.3. Prevalence of Social Sequelae Among Women of Reproductive Age

Table 2 shows the bivariate analyses of social sequelae attributable to MJ and sample characteristics in WRA with past month MJ use. The weighted prevalence of social sequelae with 95% CI and *p*-values were noted. The older WRA (26–44 years old) who used MJ in the past month had lower prevalence of social sequelae. A significantly higher proportion of women aged 18–25 years old (7.74%, 95% CI: 6.76, 8.84) had experienced social sequelae than women aged 26–44 years old (2.12%, 95% CI: 1.66, 2.71) with *p*-value < 0.001. Women with no high school education (5.89%, 95% CI: 4.59, 7.54), unemployed (8.82%, 95% CI: 6.45, 11.96), women with a health insurance gap in the past year (6.37%, 95% CI: 4.81, 8.32), women with past-year MDE (7.36%, 95% CI: 6.22, 8.68), and women with Fair/Poor self-reported overall health (5.85%, 95% CI: 4.64, 7.35) had the highest prevalence of social sequelae (*p*-value < 0.001).

### 4.4. Polysubstance Use and Social Sequelae Among Women of Reproductive Age Using Marijuana

The adjusted odds ratio (AOR) and 95% CI for social sequelae are shown in Table 3. MJ users involved in PSU or concurrent use with two forms of substance (PMU II) had higher odds of social sequelae by 68% (AOR = 1.68, 95% CI: 1.16, 2.43) than women who only used MJ in the past month (MMU) after adjusting for sociodemographic factors, and self-reported health. In addition, MJ users involved in PSU or concurrent use with three or more forms of substance (PMU III) had 3.18 times higher odds (AOR = 3.18, 95% CI: 1.90, 5.31) of social sequelae than monosubstance marijuana users (MMU) after adjusting for sociodemographic factors, and self-reported health.

Women aged 18–25 years old had 3.75 times the odds (AOR = 3.75, 95% CI: 2.67, 5.26) of social sequelae compared to women aged 26–44 years. Black, non-Hispanic women were more likely to report social sequelae (AOR = 1.76, 95% CI: 1.25, 2.50) than White, non-Hispanic women. Women who experienced MDE in the past 12 months were also more likely to report social sequelae (AOR = 1.95, 95% CI: 1.52, 2.55) compared to women who did not have any MDE in the past 12 months. Women who had a health insurance gap in the past year were more likely to report social sequelae (AOR = 1.43, 95% CI: 1.07, 1.90) compared to women who did not have any health insurance gap in the past year. Women who self-reported having Fair/Poor self-reported overall health were more likely to report social sequelae (AOR = 1.62, 95% CI: 1.14, 2.29) than women who self-reported having Excellent/Very good overall health. Lastly, women who were unemployed were more likely to report social sequelae (AOR = 1.80, 95% CI: 1.22, 2.65) than women who had full-time employment.

## 5. Discussion

Our findings show clear and consistent associations between PSU status and social sequelae when two or more substances were concurrently used with MJ in the past month timeframe. Given the increased risk of psychosocial outcomes, such as social sequelae among MJ users [8,9,10,11], the current study aimed to assess the impact of PSU status on social sequelae attributable to MJ use within the WRA population. A key aspect of this study was capturing the concurrent use of multiple substances with “overlapping substance effects” alongside MJ within the past month timeframe. This is particularly relevant for the WRA population, where preconception and interconception care is critical [3], as it addresses the unique vulnerabilities of this group. Concurrent use of multiple substances in conjunction with MJ is particularly concerning in the WRA group since women who are pregnant (or plan to be pregnant) could be affected by substance use that have been shown to adversely impact overall health of the women as well as fetal health [4,15]. In addition, our findings corroborate the notion that PSU status or concurrent use of MJ with other substances is common in the WRA group as well as the need for perinatal universal substance use screening [4,16].

Overall, this study found that younger women (18–25 years old), Black non-Hispanic women, women who reported having any MDE in the past 12 months, women who were unemployed, and women who reported using MJ with three or more substances in the past month had the highest odds of social sequelae. This finding aligns with previous research indicating that younger women are at a greater risk of experiencing social sequelae [8,17]. MJ use impairs psychosocial development and reduces the likelihood that a young woman will be able to establish a satisfactory adult life [17]. Since young women need to develop, mature, and prepare to meet demands in their adult life including childbirth and motherhood, social sequelae such as having psychosocial problems at educational and/or employment setting reduces the likelihood of young women to establish a satisfactory life [17]. However, research on racial differences in social sequelae within the general WRA population remains limited. In addition, when analyses were stratified by PSU status, concurrent use of two or more substances with MJ increased the odds of social sequelae by 68%. Lastly, this study found a MDE in the past year significantly increased the odds of social sequelae; however, reverse causality may be plausible [5], given that the past MDE timeframe was assessed in the 12-month period, whereas substance use was assessed over the past month.

This study has several limitations. Women’s substance use, including PSU status, was self-reported at the time of the interview. The NSDUH collects personal and confidential information on past month MJ use in non-institutionalized adult WRA living in the US using the ACASI method. Self-reported substance use information is not validated with biological samples. The ACASI method is designed to reduce bias by increasing honest responses related to sensitive behaviors such as reporting substance abuse or misuse [13]. Self-reported substance use and other health measures, including past year MDE and overall health, are collected in the survey [13]. However, self-reported data such as MDE or overall health may be subject to recall bias, potentially influenced by the stigmatizing behavior associated with MJ and substance use [18]. In addition, the cross-sectional design of our study precludes causal inferences and does not allow for establishing the temporal sequence between PSU status and social sequelae.

Despite these limitations, the study contributes significantly to the existing literature and extends current research in regard to PSU and psychosocial harm in WRA. It is the first study to utilize a nationally representative sample from the NSDUH to estimate social sequelae in WRA as it relates to PSU and MJ use. Findings from this study have both public health and clinical relevance, particularly in pharmacy practice. Pharmacists possess a highly specialized knowledge of medication use, safety, and efficacy, with a rapidly expanding role as it relates to patient care [19]. Pharmacy settings can act as central hubs of care for patients with drug seeking behavior [19]. For example, WRA who are visiting disparate healthcare providers to gain access to psychoactive stimulants (i.e., prescription of amphetamaine mixed salts for the treatment of attention deficit hyperactivity disorder) or cannabinoids (i.e., prescription drug such as dronabinol that contain an active ingredient also found in MJ for the treatment of appetite stimulation) can be flagged by pharmacy healthcare professionals. These healthcare providers can help prevent potential misuse and ensure that medications are prescribed and dispensed safely and appropriately. In addition to dispensing medications, pharmacists can offer counseling, education, and referrals to treatment services, making them valuable partners in prevention, early detection, and ongoing management of substance use disorders.

Given that the American College of Obstetricians and Gynecologists (ACOG), the American Academy of Pediatrics (AAP), and the World Health Organization (WHO) support universal screening for perinatal substance use due to its detrimental effects on both mothers and infants, it is imperative to expand research on polysubstance use among women of reproductive age (WRA) [3,16,20,21]. While the perinatal period offers a crucial opportunity for intervention, substance use often begins well before pregnancy and can extend far beyond the postpartum phase. This highlights the need for a broader, lifespan-oriented perspective. Clinicians and public health professionals must go beyond assessing only psychosocial outcomes and incorporate an understanding of the motivations, patterns of concurrent use of multiple substances, and the social and structural factors influencing substance use throughout the reproductive years.

## Figures and Tables

**Table 1:** Sample characteristics of women of reproductive age (18–44 years old) who used marijuana in the past month stratified by polysubstance use status.

Characteristics	Total N = 11,895 Weighted % (95% CI)	Monosubstance Marijuana Users (MMU) * N = 2770 Weighted % (95% CI)	Polysubstance Marijuana Users I (PMU I) ** N = 5129 Weighted % (95% CI)	Polysubstance Marijuana Users II (PMU II) *** N = 3142 Weighted % (95% CI)	Polysubstance Marijuana Users III (PMU III) **** N = 854 Weighted % (95% CI)	*p*-Value
**Social Sequelae (SS)**						<0.001
No	95.55 (95.02, 96.02)	96.19 (95.19, 96.98)	96.46 (95.87, 96.98)	95.04 (93.82, 96.03)	89.82 (86.55, 92.37)	
Yes	4.45 (3.98, 4.98)	3.81 (3.02, 4.81)	3.54 (3.02, 4.13)	4.96 (3.94, 6.18)	10.18 (7.63, 13.45)	
**Age**						0.0467
18–25 years	41.65 (40.65, 42.66)	42.54 (40.07, 45.05)	41.32 (39.72, 42.94)	40.09 (38.27, 41.94)	46.55 (42.21, 50.94)	
26–44 years	58.35 (57.34, 59.35)	57.46 (54.95, 59.93)	58.68 (57.06, 60.28)	59.91 (58.06, 61.73)	53.45 (49.06, 57.79)	
**Race/Ethnicity**						<0.001
White non-Hispanic	61.66 (60.14, 63.16)	53.51 (51.01, 55.99)	62.43 (60.20, 64.60)	64.41 (62.07, 66.69)	73.23 (68.94, 77.12)	
Black non-Hispanic	16.11 (15.08, 17.19)	22.13 (20.25, 24.12)	15.50 (14.10, 17.01)	14.44 (12.64, 16.45)	6.44 (4.55, 9.03)	
Asian/other non-Hispanic	7.87 (7.12, 8.70)	9.08 (7.62, 10.78)	7.57 (6.58, 8.68)	7.22 (5.89, 8.81)	8.26 (6.51, 10.44)	
Hispanic	14.36 (13.46, 15.31)	15.28 (13.74, 16.98)	14.50 (13.17, 15.95)	13.93 (12.59, 15.39)	12.07 (9.83, 14.74)	
**Education**						0.023
No high school	9.48 (8.84, 10.15)	8.37 (7.12, 9.83)	9.46 (8.40, 10.64)	10.19 (8.85, 11.71)	10.53 (8.01, 13.74)	
High school or GED	22.53 (21.47, 23.61)	21.45 (19.07, 24.03)	21.62 (19.95, 23.38)	25.36 (23.47, 27.35)	21.02 (18.14, 24.22)	
Some college/College graduate	67.99 (66.91, 69.06)	70.18 (67.92, 72.34)	68.92 (67.17, 70.63)	64.45 (62.45, 66.41)	68.45 (63.86, 72.70)	
**Employment status**						<0.001
Full-time	49.29 (48.22, 50.37)	48.08 (45.39, 50.78)	51.28 (49.83, 52.73)	47.90 (45.48, 50.33)	46.38 (42.06, 50.75)	
Part-time	21.89 (21.09, 22.70)	23.84 (21.77, 26.04)	21.34 (20.14, 22.58)	21.09 (19.33, 22.98)	21.80 (18.25, 25.83)	
Unemployed	7.77 (7.13, 8.46)	5.83 (4.93, 6.89)	6.88 (5.96, 7.97)	9.35 (8.20, 10.71)	13.46 (10.82, 16.61)	
Other	21.05 (20.01, 22.13)	22.25 (20.54, 24.06)	20.50 (18.98, 22.08)	21.66 (19.70, 23.74)	18.36 (15.56, 21.54)	
**Health insurance gap in the past year**						0.002
Yes	11.01 (10.15, 11.93)	10.86 (9.34, 12.58)	9.60 (8.61, 10.69)	12.38 (10.77, 14.18)	15.37 (11.67, 19.98)	
No	88.99 (88.07, 89.85)	89.14 (87.42, 90.66)	90.4 (89.31, 91.39)	87.62 (85.82, 89.23)	84.63 (80.02, 88.33)	
**Past-year major depressive episode**						<0.001
Yes	22.59 (21.63, 23.58)	21.76 (19.92, 23.73)	20.48 (19.22, 21.79)	24.93 (22.94, 27.03)	29.49 (25.45, 33.88)	
No	77.41 (76.42, 78.37)	78.24 (76.27, 80.08)	79.52 (78.21, 80.78)	75.07 (66.12, 74.55)	70.51 (66.12, 74.55)	
**Self-reported overall health**						<0.001
Excellent/Very good	56.13 (54.79, 57.46)	59.33 (56.30, 62.28)	58.06 (56.21, 59.88)	50.60 (48.22, 52.99)	54.61 (50.10, 59.05)	
Good	29.49 (28.23, 30.79)	27.45 (24.94, 30.12)	28.57 (26.82, 30.38)	33.33 (30.87, 35.89)	27.52 (23.57, 31.86)	
Fair/Poor	14.38 (13.51, 15.29)	13.22 (11.36, 15.35)	13.37 (12.08, 14.79)	16.07 (14.42, 17.86)	17.87 (14.60, 21.68)	

* MMU refers to women involved in monosubstance use of marijuana only in the past month without use of other substances (alcohol binge drinking, opioids, psychoactive stimulants, and tobacco). ** PMU I refers to women involved in polysubstance use of marijuana in the past month with one other substance (alcohol binge drinking, opioids, psychoactive stimulants, or tobacco). *** PMU II refers to women involved in polysubstance use of marijuana in the past month with two other substances (alcohol binge drinking, opioids, psychoactive stimulants, or tobacco). **** PMU III refers to women involved in polysubstance use of marijuana in the past month with three or more other substances (alcohol binge drinking, opioids, psychoactive stimulants, or tobacco).

**Table 2:** Prevalence of social sequelae among women of reproductive age (18–44 years old) who used marijuana in the past month.

Characteristics	Social Sequelae (SS)		*p*-Value
	No SS Weighted Prevalence (95% CI)	Yes SS Weighted Prevalence (95% CI)	
**Age**			<0.001
18–25 years	92.26 (91.16, 93.24)	7.74 (6.76, 8.84)	
26–44 years	97.88 (97.29, 98.34)	2.12 (1.66, 2.71)	
**Race/Ethnicity**			0.002
White non-Hispanic	96.29 (95.76, 96.76)	3.71 (3.24, 4.24)	
Black non-Hispanic	94.23 (92.40, 95.64)	5.77 (4.36, 7.60)	
Asian/other non-Hispanic	94.40 (92.17, 96.02)	5.60 (3.98, 7.83)	
Hispanic	94.43 (92.99, 95.58)	5.57 (4.42, 7.01)	
**Education**			0.001
No high school	94.11 (92.46, 95.41)	5.89 (4.59, 7.54)	
High school or GED	94.29 (93.12, 95.27)	5.71 (4.72, 6.88)	
Some college/College graduate	96.16 (95.49, 96.74)	3.84 (3.26, 4.51)	
**Employment status**			<0.001
Full-time	96.67 (96.00, 97.24)	3.33 (2.76, 4.00)	
Part-time	93.95 (92.74, 94.97)	6.05 (5.03, 7.26)	
Unemployed	91.18 (88.04, 93.55)	8.82 (6.45, 11.96)	
Other	96.17 (95.25, 96.92)	3.83 (3.08, 4.75)	
**Health insurance gap in the past year**			0.003
Yes	93.63 (91.68, 95.14)	6.37 (4.81, 8.32)	
No	95.72 (95.19, 96.19)	4.28 (3.81, 4.81)	
**Past-year major depressive episode**			<0.001
Yes	92.64 (91.32, 93.78)	7.36 (6.22, 8.68)	
No	96.44 (95.91, 96.90)	3.56 (3.10, 4.09)	
**Self-reported overall health**			<0.001
Excellent/Very good	96.55 (95.96, 97.05)	3.45 (2.95, 4.04)	
Good	94.32 (93.29, 95.20)	5.68 (4.80, 6.71)	
Fair/Poor	94.15 (92.65, 95.36)	5.85 (4.64, 7.35)	

**Table 3:** Associations between social sequelae and sample characteristics in women of reproductive age (18–44 years old) who used marijuana in the past month stratified by polysubstance use status.

Characteristics	Social Sequelae Adjusted Odds Ratio	Social Sequelae Adjusted Odds Ratio (95% CI)	*p*-Value
**Polysubstance Use Status**			
Monosubstance Marijuana Users (MMU) *	Reference	Reference	
Polysubstance Marijuana Users I (PMU I) **	1.12	(0.81, 1.56)	0.48
Polysubstance Marijuana Users II (PMU II) ***	1.68	(1.16, 2.43)	0.007
Polysubstance Marijuana Users III (PMU III) ****	3.18	(1.90, 5.31)	<0.001
**Age**			
18–25 years	3.75	(2.67, 5.26)	<0.001
26–44 years	Reference	Reference	
**Race/Ethnicity**			
White non-Hispanic	Reference	Reference	
Black non-Hispanic	1.76	(1.25, 2.50)	<0.001
Asian/other non-Hispanic	1.37	(0.88, 2.21)	0.16
Hispanic	1.29	(0.96, 1.74)	0.085
**Education**			
No high school	1.21	(0.84, 1.74)	0.31
High school or GED	1.19	(0.90, 1.59)	0.085
Some college/College graduate	Reference	Reference	
**Employment status**			
Full-time	Reference	Reference	
Part-time	1.60	(1.15, 2.23)	0.006
Unemployed	1.80	(1.22, 2.65)	0.004
Other	1.01	(0.72, 1.44)	0.93
**Health insurance gap in the past year**			
Yes	1.43	(1.07, 1.90)	0.016
No	Reference	Reference	
**Past-year major depressive episode**			
Yes	1.97	(1.52, 2.55)	<0.001
No	Reference	Reference	
**Self-reported overall health**			
Excellent/Very good	Reference	Reference	
Good	1.69	(1.32, 2.16)	<0.001
Fair/Poor	1.62	(1.14, 2.29)	0.008

* MMU refers to women involved in monosubstance use of marijuana only in the past month without use of other substances (alcohol binge drinking, opioids, psychoactive stimulants, and tobacco). ** PMU I refers to women involved in polysubstance use of marijuana in the past month with one other substance (alcohol binge drinking, opioids, psychoactive stimulants, or tobacco). *** PMU II refers to women involved in polysubstance use of marijuana in the past month with two other substances (alcohol binge drinking, opioids, psychoactive stimulants, or tobacco). **** PMU III refers to women involved in polysubstance use of marijuana in the past month with three or more other substances (alcohol binge drinking, opioids, psychoactive stimulants, or tobacco).

## Data Availability

The NSDUH data is publicly available data or public use files through the Substance Abuse and Mental Health Services Administration website. Files are available for download allowing researches and the public to analyze data on substance use and mental health.

## Abbreviations

The following abbreviations are used in this manuscript:

PSU	Polysubstance use
MJ	Marijuana
WRA	Women of reproductive age
NSDUH	National Survey on Drug Use and Health
ACASI	Audio computer-assisted self-interviewing
PMU	Polysubstance marijuana users
MMU	Monosubstance marijuana users
MDE	Major depressive episode
ACOG	American College of Obstetricians and Gynecologists
WHO	World Health Organization
AAP	American Academy of Pediatrics
